# Sorting mRNA Molecules for Cytoplasmic Transport and Localization

**DOI:** 10.3389/fgene.2018.00510

**Published:** 2018-11-06

**Authors:** Nathalie Neriec, Piergiorgio Percipalle

**Affiliations:** ^1^Biology Department, New York University Abu Dhabi, Abu Dhabi, United Arab Emirates; ^2^Department of Molecular Biosciences, The Wenner-Gren Institute, Stockholm University, Stockholm, Sweden

**Keywords:** mRNA transport and localization, hnRNP proteins, protein-RNA binding, G4 quadruplex, oligodendrocytes, neurons, spermatogenic cells

## Abstract

In eukaryotic cells, gene expression is highly regulated at many layers. Nascent RNA molecules are assembled into ribonucleoprotein complexes that are then released into the nucleoplasmic milieu and transferred to the nuclear pore complex for nuclear export. RNAs are then either translated or transported to the cellular periphery. Emerging evidence indicates that RNA-binding proteins play an essential role throughout RNA biogenesis, from the gene to polyribosomes. However, the sorting mechanisms that regulate whether an RNA molecule is immediately translated or sent to specialized locations for translation are unclear. This question is highly relevant during development and differentiation when cells acquire a specific identity. Here, we focus on the RNA-binding properties of heterogeneous nuclear ribonucleoproteins (hnRNPs) and how these mechanisms are believed to play an essential role in RNA trafficking in polarized cells. Further, by focusing on the specific hnRNP protein CBF-A/hnRNPab and its naturally occurring isoforms, we propose a model on how hnRNP proteins are capable of regulating gene expression both spatially and temporally throughout the RNA biogenesis pathway, impacting both healthy and diseased cells.

## Introduction

A fascinating question in gene expression regulation is to understand how from the onset of transcription, cells regulate mRNA molecules into degradation, localization, storage, and/or translation. Several decades of mRNA biology have shown that regulation primarily happens at the level of ribonucleoprotein (RNP) particles, composed of RNA molecules and RNA-Binding Proteins (RBPs) (Dreyfuss et al., 2002). Within RNP particles, the protein composition evolves as the RNA is synthesized and matured. Different sets of RBPs join nascent RNP particles at specific steps of mRNA synthesis and maturation, such as splicing or nuclear export, others accompany the mRNA from the onset of transcription all the way to translation. One of the most intriguing aspects is, therefore, to understand how and why protein-RNA interactions are established from gene to polyribosomes (or polysomes), whether and how they lead to specific fates for the mRNA.

In this mini-review, we concentrate on two key steps in the mRNA regulation by focusing on a representative of a large family of RBPs, the heterogeneous nuclear ribonucleoprotein ab (hnRNPab) also referred to as CBF-A (CArG box-binding factor A). After a brief review of the different stages of mRNA biogenesis, we will address the role of hnRNPab in the formation and integrity of RNP particles, and in the regulation of the translatability of the carried mRNA. Finally, we will discuss the relevance of those mechanisms in cell specification and development.

## mRna Biogenesis From the Gene to Polysomes

### Nascent Transcripts and Nuclear Organization

**FIGURE 1:**
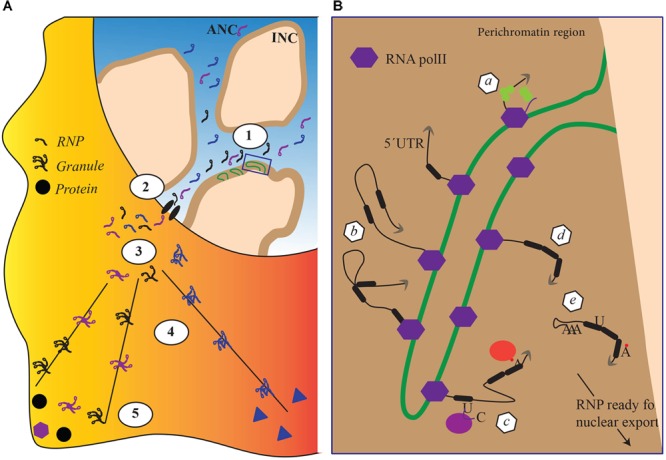
The impact of nuclear architecture on RNA biogenesis. **(A)** Transcriptional and co transcriptional events (1), nuclear export (2), granule formation (3), transport and translation repression (4) local anchoring and translation derepression (5). Each step corresponds to a re-organization of the RBPs attached to the RNA. **(B)** Magnification of detail within box, schematically representing nuclear co transcriptional events leading to the mature RNPs competent for export. Each event schematically represented in panel **B** depends on different sets of RBPs and lead to a distinctive, final RBP composition within the RNP. (a) capping, (b) splicing, (c–d) RNA editing and RNA modifications, and (e) cleavage and addition of a poly(A)tail.

mRNA biogenesis is fundamentally affected by the organization of the cell nucleus. During differentiation, tissue-specific promoters are switched on or off to consolidate specific cellular identities and this coincides with changes in the localization of genes within the nucleus. Actively transcribed genes are believed to be located in a chromosome domain that borders with interchromosomal spaces, the perichromatin region. In that region, gene-rich chromosome loops, characterized by decondensed chromatin, project into the DNA-depleted interchromosomal space (Cremer et al., 2015; Figure 1). Although not all studies agree with the existence of the interchromatin space (Branco and Pombo, 2006), work on the polytene chromosome from the dipteran insect *Chironomus tentans* has shown *in situ* evidence of RNP particles decorating chromosome loops and being released after the maturation in the interchromatin space (Daneholt, 1997; Daneholt, 2001; Percipalle et al., 2001). Recently, Cremer et al. (2015) reviewing all literature from imaging to electron microscopy proposed a formalized nomenclature for the architectural organization of the nucleus. In the model, there are two coaligned three-dimensional networks termed Active and Inactive Nuclear Compartments (ANC and INC, respectively) (Cremer et al., 2015; Hübner et al., 2015). The INC contains the silenced chromatin, whereas the ANC, divided in the perichromatin and the interchromosomal space, contains the active DNA regions. In this model, the nucleus is represented as a sponge-like structure where the INC is perforated with channels of interchromosomal space connecting adjacent nuclear pores. The linings of those channels constitute the perichromatin regions where the contents of the interchromosomal space (including transcription factors and RBPs) can interact with the active unpacked DNA (Cremer et al., 2015; Hübner et al., 2015; Figure 1).

In the above model, the perichromatin region becomes its own nuclear subcompartment where transcription and cotranscriptional events take place (Figure 1B), acting as a hub for chromatin remodelers and histone-modifying enzymes to maintain an open chromatin state required for transcription. At the onset of transcription, nascent transcripts exiting the RNA polymerase machinery promote recruitment of RBPs. Among RBPs, hnRNP proteins are believed to be among the first ones to bind the nascent transcript, protecting it from degradation and facilitating cotranscriptional RNP assembly. The protein composition of an RNP particle depends on the specific mRNA, cell type, and stage and is remodeled throughout mRNA capping, splicing, cleavage, and polyadenylation (Figure 1B; for review see Singh et al., 2015). At the end of transcription newly formed RNP particles are released in interchromatin spaces. The initial steps in the biogenesis of RNP particles, in particular cotranscriptional RNP particle assembly, are therefore exquisitely integrated into the architecture of the cell nucleus. However, how this integration is maintained within the perichromatin region while particles move on the chromatin loop is unclear. Most likely, RNP particles are somehow connected to the chromatin as the mRNA is transcribed to protect it from being pulled into the interchromosomal space. The mechanisms by which such flexible anchoring could happen are unknown. Although their existence is not fully proven, transcription factories – where polymerases remain anchored and the DNA moves through the factory itself – may play an important role in maintaining nascent RNP particles connected to the chromatin but in this case the RNP particle would be a relatively static entity (Sutherland and Bickmore, 2009).

### From the Gene to Polysomes, Sorting Transcripts for Localized Translation

In the interchromatin space, mature RNP particles are believed to migrate by passive diffusion toward the nuclear envelope (Singh et al., 1999; Shav-Tal et al., 2004). Once at the nuclear pore complex (NPC), RNP particles are exported, a process that is considerably more rapid than the passive diffusion across the nucleoplasm (Bjork and Wieslander, 2017). As the RNP particle is routed toward the NPC, its composition changes with certain proteins being dynamically added or shed away from the transcript (Dreyfuss et al., 2002; Oeffinger and Montpetit, 2015). This fundamentally affects the intrinsic properties of the RNP particle. For instance, work performed by electron microscopy in *C. tentans* demonstrated that RNP particles unfold as they pass through the NPC, exposing the 5′ end for immediate translation on the polysomes (Daneholt, 1997, 2001). In mammals, probably not all RNP particles completely unfold during passage through the NPC. However, RNP particles clearly transition from a highly compact macromolecular assembly to a more loosely organized entity, demonstrating a considerable degree of intrinsic plasticity. Although the mechanisms are not fully understood, remodeling of the mRNA molecule performed by RNA helicases in combination with changes in the polymerization state of actin has been suggested to be the driving forces (Figure 1B; Percipalle et al., 2001; Percipalle, 2014).

All RNP particles are not immediately translated as they exit from the nucleus. A subset of RNP particles is transported to cellular compartments where they are either stored in a translationally inactive form or locally translated. Examples of sites where transcripts are stored are provided by transport granules in neurites (reviewed in Lee et al., 2016) and chromatoid bodies in spermatogenic cells (Kotaja and Sassone-Corsi, 2007). To reach specialized sites for local translation, transcripts are rapidly transported. In polarized cells such as neurons and oligodendrocytes there are several well-studied examples of transcripts being transported to dendrites and myelin compartment, respectively (Martin and Ephrussi, 2009). Although the mechanisms are not fully understood, prior to transport to such specialized locations, RNP particles are believed to assemble into large granules that probably contain many copies of the same transcript and are actively transported via the microtubule system (Figure 1A; for reviews see Carson and Barbarese, 2005; Kindler et al., 2005). Cytoplasmic RNA transport requires specific *cis*-acting elements within the mRNA termed zip codes that are presented to cellular transacting factors such as RBPs. These interactions are likely to stabilize transport-competent RNP particles and possibly, the formation of granules that are then transported to their final cytoplasmic destinations where the mRNA is released and localized for translation. All these mechanisms require several coordinated steps that are not fully understood.

## The Involvement of CBF-A/hnRnpab in RNPs Assembly, Transport, and Localization

**FIGURE 2:**
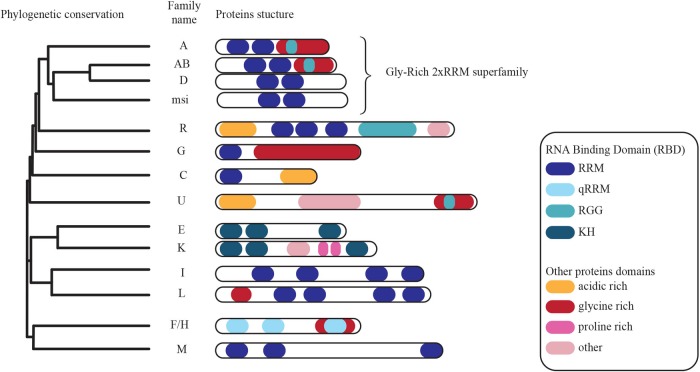
Phylogenetic conservation of members of the heterogeneous nuclear ribonucleoprotein family of proteins. Conserved functional domains are highlighted (modified from Geuens et al., 2016).

A central question is at what stage and which *cis*-acting elements are targeted by specific cellular transacting factors to regulate the different layers of RNA biogenesis. A good example is provided by the hnRNP protein CBF-A/hnRNPab that is known to interact with several RNAs through a *cis*-acting element termed RNA trafficking sequence (RTS) or A2 Response Element (A2RE), in order to regulate cytoplasmic mRNA transport (Raju et al., 2008, 2011; Fukuda et al., 2013). CBF-A/hnRNPab was identified as a single-stranded DNA-binding protein interacting with CarG boxes, CC(A/T-rich)6GG, present in the α-Smooth Muscle Actin (Kamada and Miwa, 1992) and several others including apoVLDII and RSV CarG-boxes (Smidt et al., 1995), Ig κ promoter (Bemark et al., 1998), Arginine VasoPressine (AVP) (Murgatroyd et al., 2004). We showed that CBF-A/hnRNPab also binds to poly(A) mRNA *in vitro* and in living cells (Percipalle et al., 2002). From an evolutionary point of view, CBF-A/hnRNPab actually belongs to the conserved hnRNP subfamily of the “2^∗^RNA Binding Domain (RBDs) and Glycine-rich auxiliary domain” (2^∗^RBD-Gly) proteins (Aranburu et al., 2006; Figure 2). As all 2^∗^RBD-Gly proteins, CBF-A/hnRNPab is composed of a unique non-conserved N-terminal region, a highly conserved central region that contains two RNA-binding domains (RBDs) and a conserved C-terminal Glycine-rich region (Dreyfuss et al., 1993; Smidt et al., 1995; Lau et al., 1997; Rushlow et al., 1999, 2000; Weisman-Shomer et al., 2002; Khateb et al., 2004; Aranburu et al., 2006). The closest homolog to CBF-A/hnRNPab, hnRNPD is also a member of the 2^∗^RBD-Gly family together with hnRNPA0 to A3 and Musashi (Aranburu et al., 2006). CBF-A/hnRNPab and the other members of the 2^∗^RBD-Gly family undergo remarkably similar alternative splicing, which generates different proteins differing by just few kilodaltons (Dean et al., 2002; Kroll et al., 2009; Gueroussov et al., 2017). Conserved among mammals, CBF-A/hnRNPab has two isoforms, p37 (284 amino acids) and p42 (331 amino acids) (Khan et al., 1991; Lau et al., 1997; Yabuki et al., 2001). The two isoforms p37 and p42 have been shown to have different RNA and DNA-binding properties, they bind to different proteins and appear to have different roles in the cell (Yabuki et al., 2001; Fomenkov et al., 2003; Fukuda et al., 2013). Both the isoforms have been located in the nucleus and in the cytoplasm within RNA granules and they appear to be functionally different in the context of RNA regulation. For instance, the p42 isoform, but not p37, is involved in alternative splicing via binding of the specific α sterile motif of the p53 family member p63α. This interaction regulates alternative splicing of the *Fgfr2* mRNA from a mesenchymal form to an epithelial form. Suppression of the CBF-A/hnRNPab-p63α interaction has been suggested to be the cause of craniofacial disorders such as the Hay-Wells syndrome (Fomenkov et al., 2003). Such protein-protein interactions are also known to lead to the production of the dominant negative mRNA isoforms α and β of the *Tert* telomerase (Vorovich and Ratovitski, 2008). However, the molecular mechanisms by which the hnRNPab-p63α interaction affects mRNA splicing are not understood. Furthermore, there is evidence that CBF-A/hnRNPab is involved in *ApoB* editing by recruiting APOBEC1 and possibly disrupts the secondary structure of *ApoB* mRNA. Whether both the isoforms are similarly engaged in the process remain to be elucidated (Lau et al., 1997).

Here, we will focus on how the two CBF-A/hnRNPab isoforms have been suggested to be involved in the regulation of RNP particles from the onset of transcription in the nucleus to the mRNAs translatability upon transport.

### Sorting Transport-Competent RNA Occurs During Nuclear Preparatory Events

CBF-A/hnRNPab is among the RBPs that seem to interact with the transcript at an early stage during the RNA biogenesis pathway. In fact, in thin sections of adult mouse brain, antibodies to CBF-A/hnRNPab decorated electrodense structures located in the interchromosomal space and in the perichromatin area (Raju et al., 2011), where active transcription takes place (Fakan and Puvion, 1980). In contrast the same antibodies to CBF-A/hnRNPab did not stain patches of dense chromatin. Based on location and morphology, CBF-A/hnRNPab seems to be excluded from INC while it is enriched at the ANC compartment associating with (pre)-mRNP complexes at sites of transcription and in the interchromosomal space. In the same study, CBF-A/hnRNPab was also found to be associated with electrodense structures, presumably mRNP particles, passing through the nuclear pores and in transit to the cytoplasm (Raju et al., 2011; Fukuda et al., 2013). Therefore, seeing that CBF-A/hnRNPab binds to poly(A) mRNA, it seems conceivable that CBF-A/hnRNPab cotranscriptionally associates with the transcripts and accompanies them to the cytoplasm. We speculate that binding of specific RBPs to nascent transcripts is a way of sorting them for specialized functions and CBF-A/hnRNPab may perform this specific task through its specific interaction with the RTS sequence.

Insights into sequence-specific recognition of single-stranded nucleic acids by hnRNP proteins came from the crystal structure of the two RNA-binding motifs (RRM) of hnRNP A1 in complex with single-stranded guanine-rich telomeric DNA. Guanine-rich DNA and RNA sequences have a tendency to form tetrahelical G4-quadruplex structures *in vitro* and *in vivo*, which appear to be stabilized by the cooperative interactions of the two hnRNP A1 molecules (Ding et al., 1999). Although not all RNAs form a G4-quadruplex, this mode of binding may explain how specific hnRNP-RNA interactions are established. For instance, similarly to single-stranded guanine-rich telomeric DNA, the hnRNPab sequence target, the CarG boxes, contains clusters of adjacent guanine residues. Furthermore, CBF-A/hnRNPab interacts with and can either disrupt the DNA quadruplex structure-like in the case of the d(CGG)n repeats in the *Fmrp1* 3′ UTR region or destabilize quadruplexes formed by the sequence [d(TTAGGG)n] at telomeres (Sarig et al., 1997a,b; Weisman-Shomer et al., 2002). These binding properties are conserved among the members of the 2^∗^RBD Gly hnRNP protein family. In fact, hnRNPA2, similarly to CBF-A/hnRNPab, also interacts with *Fmrp1* DNA quadruplexes and destabilizes the quadruplex structure. In addition, hnRNPA2 and CBF-A/hnRNPab bind r(CGG) quadruplexes. However, while hnRNPA2 efficiently disrupts such a structure, CBF-A/hnRNPab has the opposite effect and stabilizes the RNA G4-quadruplexes (Weisman-Shomer et al., 2000, 2002; Khateb et al., 2004). While the role of RNA quadruplexes is still unclear, more and more proteins involved in their recognition, folding, and unfolding are being isolated. Conserved quadruplex forming sequences have been shown to be enriched at telomeres, origin of replication, promoter region, within RNA transcripts at 3′ and 5′ UTR as well as spliced introns (Rhodes and Lipps, 2015). Not only are DNA and RNA G4 quadruplexes believed to be involved in the regulation of transcription and RNA processing (Rhodes and Lipps, 2015), but more and more studies suggest that RNA G4 quadruplexes could have an essential role in the control of translation (Song et al., 2016).

**FIGURE 3:**
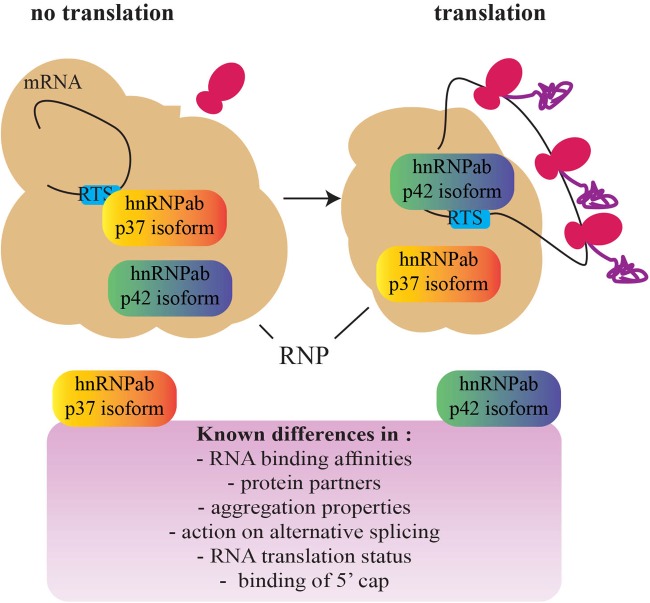
A schematic representation of the switch between the hnRNPab isoforms during the establishment of a translationally competent RNP particle. Based on the different properties of the two CBF-A/hnRNPab isoforms, a model has been proposed for translation derepression of Protamine 2 mRNA during development of spermatogenic cells (adapted from Fukuda et al., 2013). A fascinating question that remains to be addressed is whether this mechanism is found in other cell types and impacts development (RNP, ribonucleoprotein; RTS, RNA trafficking sequence).

With this in mind, CBF-A/hnRNPab may cotranscriptionally target *cis*-acting elements within nascent RNA and stabilize the formation of RNA G4 quadruplexes to sort transcripts that are not translationally active and can therefore be transported to the cellular periphery. Indeed, CBF-A/hnRNPab binds to the RTS located in the 3′ UTR of several transcripts, including the Myelin Basic Protein (MBP), β-actin, Arc, BDNF, CAMKIIα, and Protamine 2 mRNAs (Ainger et al., 1997; Czaplinski et al., 2005; Czaplinski and Mattaj, 2006; Raju et al., 2008, 2011; Kroll et al., 2009; Fukuda et al., 2013; Andreou et al., 2014). RTS binding by CBF-A/hnRNPab is required for transport and localization of all of the above transcripts to the cellular periphery where they are translated and deletion studies by siRNA or gene knockout have demonstrated impaired RTS-dependent mRNA transport in oligodendrocytes, neurons, and spermatogenic cells to specific cellular locations (reviewed in Percipalle, 2014). The RTS element is recognized by other members of the 2^∗^RBD-Gly-rich family such as hnRNPA2 (Hoek et al., 1998) and hnRNPA3 (Ma et al., 2002). CBF-A/hnRNPab, however, seems to exhibit a higher RTS-binding affinity, at least *in vitro* (Fukuda et al., 2013). Given that RTSs are guanine-rich sequences, we speculate that RTS binding primarily by CBF-A/hnRNPab may result in a stable RNA secondary structure reminiscent of RNA quadruplexes that may require synergy with other RTS-binding hnRNP proteins. We hypothesize that this stabilization leads to a translationally repressed form of the transcript. CBF-A/hnRNPab, by interacting to the RTS of nascent RNA molecules, may regulate their translatability at a cotranscriptional stage and contribute to sort transcripts for cytoplasmic transport and localization at an early stage during the gene expression process (see Figure 3).

### Cytoplasmic Transport Granules and Their Final Destinations

As mentioned above, upon nuclear export, translationally repressed RNPs are further assembled into larger granules to be transported to specific cellular locations for storage or for translation. Although poorly understood, assembly of RNP particles into transport granules has been proposed to be mediated by homo-dimerization of RNA-bound hnRNPs and actin polymerization from within individual RNP particles (Kanai et al., 2004; Carson and Barbarese, 2005; Percipalle, 2014). The homodimerization model is in line with the idea that granules are believed to contain only one type of mRNA and a specific set of RBPs (Sinnamon and Czaplinski, 2011). CBF-A/hnRNPab, bound to the RTS element, may be important for granule formation as there is evidence that it preferentially homo-dimerizes in the cytoplasm and directly interacts with actin within the RNP particle (Percipalle et al., 2002; Aranburu et al., 2006). In addition, CBF-A/hnRNPab, similarly to hnRNP D, is present in several RNA granules, including Stau2, Btz (Fritzsche et al., 2013), kif5a (Kanai et al., 2004; Elvira et al., 2006), imp (Jonson et al., 2007), IMP1 (Weidensdorfer et al., 2009), hmm A3G (Chiu et al., 2006), RNA granules but not within the RNP granules of Stau1 (Brendel et al., 2004), and nor Ago1 and Ago2 (Hock et al., 2007).

How RNA transcripts become available to the translation machinery remains a major question for future studies. Insights recently came from evidence of different roles performed by the CBF-A/hnRNPab isoforms in regulating translatability of the Protamine 2 mRNA (Figure 3). While both the isoforms interact with the same RTS sequence, *in vitro* p37 shows a higher affinity than p42 and hnRNPA2 for the same RTS target (Fukuda et al., 2013). p42 and hnRNPA2 both interact with the RTS and 5′ Cap-binding complex. In contrast, although p37 tightly binds to the RTS it does not interact with the 5′ Cap-binding complex (Fukuda et al., 2013; Tcherkezian et al., 2014). Furthermore, both the isoforms are in the RNP (Kumar et al., 1987; Percipalle et al., 2002; Czaplinski et al., 2005; Fukuda et al., 2013) but only p42 interacts with the Protamine 2 mRNA when it is associated with translating polysomes (Fukuda et al., 2013). Altogether, these observations suggest that p37 and p42 binding to the RTS facilitate remodeling or structural disruptions of the RNP particle/granule, exposing the transcript to different molecular machinery and leading to translation (Fukuda et al., 2013). A similar “switch” between RBPs has been shown to happen on the Cox2 mRNA in macrophages where the RBP Tristetraprolin TTP is replaced by HuR once at destination (Tiedje et al., 2012). In addition, in oligodendrocytes, hnRNPA2 phosphorylation leads to the replacement of the translation repressor hnRNPE1 with the activator hnRNPK (Muller et al., 2013; Torvund-Jensen et al., 2014). Further studies will possibly address if switch mechanisms are general or transcript-specific and if other hnRNPs such as hnRNPD cooperate with the p37/p42 isoforms.

One of the open questions is how differential RTS binding of the two isoforms is achieved. Recently, hnRNPA2 has been shown to be involved in alternative splicing of miRNAs by recognizing methylation on adenosine residues (Alarcón et al., 2015). Since both CBF-A/hnRNPab and hnRNPA2 bind the same RTS site (Fukuda et al., 2013), it is intriguing to speculate that the methylation state of the RTS sequence may be involved in the binding affinity of CBF-A/hnRNPab, promoting binding of p37 or p42 together with other hnRNP proteins. Whether and how all of the above mechanisms in a coordinated manner lead to optimal RNP particle remodeling at different stages of RNA biogenesis remains, however, to be understood. Some of these questions may become clearer once we understand the full spectrum of protein modifications involved and if RNA methylation plays a role in regulating differential RNA-binding affinities.

### CBF-A/hnRNPab Regulation of Cell Specification and Development

The mechanisms of RNA trafficking are important to ensure spatial and temporal regulation of gene expression, which is, in turn, required during development and differentiation. Understanding how the CBF-A/hnRNPab isoforms promote efficient mRNA trafficking might, therefore, provide an interesting paradigm to study cell specification and neuronal development. Indeed, high levels of CBF-A/hnRNPab expression can be found in neuronal cells, in the developing neural tissues and neurogenic regions of the brains (Rushlow et al., 1999; Gong et al., 2003) In *Xenopus laevis*, depletion of CBF-A/hnRNPab orthologs led to a decrease in eye size due to a general increase in apoptosis, as well as a decrease in proliferative neural tissues, with cranial neurons not being properly formed, motor neurons missing and defects in migration. Depleted neurons also show a thinner and disorganized tubulin network (Andreou et al., 2014). In mice, neurospheres produced from CBF-A/hnRNPab^−/−^ knock-out mice have reduced expression in the stem cell marker Nestin and an increase in the differentiated marker dcx. This suggests that CBF-A/hnRNPab is involved in the regulation of stem cell maintenance and neuronal precursor differentiation. Furthermore, CBF-A/hnRNPab^−/−^ neurons *in vivo* have neurites length increased by 40% while their longest neurite is 32% longer than the wild-type condition (Sinnamon et al., 2012). Finally, nerve growth stimulation resulted in increased CBF-A/hnRNPab expression (Rushlow et al., 1999, 2000). How CBF-A/hnRNPab^−/−^ is involved in neuronal development is not known. In both neuronal cells and spermatogenic cells CBF-A/hnRNPab was found to interact with the 5′ Cap-binding complex, facilitating translation (Fukuda et al., 2013; Tcherkezian et al., 2014). An interesting possibility is that the p37–p42 relay mechanism might be important to translationally repress and/or derepress transcripts that are important for neuronal development.

The above mechanisms are likely to also occur in the adult brain since CBF-A/hnRNPab is expressed in mature neurons, oligodendrocytes, and astrocytes (Rushlow et al., 1999). Lack of CBF-A/hnRNPab in cultured oligodendrocytes results in impaired transport and localization of MBP mRNA at the myelin compartment (Raju et al., 2008). In primary neurons, localization of CBF-A/hnRNPab at postsynaptic compartments is enhanced by the treatment with NMDA and AMPA (Raju et al., 2011), suggesting an activity-dependent role for CBF-A/hnRNPab. Furthermore, CBF-A/hnRNPab has been shown to repress *in vivo* excitotoxicity, a phenomenon that is a direct consequence of over stimulation of glutamatergic neurons that can lead to cell stress and neuronal cell death (Sinnamon et al., 2012). Interestingly, hypersensitivity to excitotoxicity revealed in hnRNPab-/- glutamatergic neurons has been proposed as a mechanism involved in neurodegenerative disorder (Lau and Tymianski, 2010). Consistently, emerging evidence suggests that the members of the 2^∗^RBD-Gly family mimic Alzheimer’s disease phenotypes at the cellular level when the proteins are depleted *in vitro* (Berson et al., 2012). In light of these observations, a fascinating avenue to be explored in the future would be to find out whether there is a connection between suppression of specific 2^∗^RBD-Gly family members and the onset of neurodegenerative disorders.

## Conclusion

We suggest that the mechanisms described above based on the ubiquitously expressed CBF-A/hnRNPab isoforms overall contribute to the general translatability of mRNA transcripts. This is initially achieved in the cell nucleus; we propose that at this stage nascent transcripts are translationally repressed and consequently sorted for cytoplasmic transport and localization (Figure 3). The contribution of chromatin at this stage remains to be understood. Once they reach their final cytoplasmic location, however, transcripts are translationally derepressed. Control of translatability may be achieved by a relay mechanism based on the different RNA-binding affinities of the two CBF-A/hnRNPab isoforms. Different binding affinities may be a consequence of RNA methylation. Although much remains to be uncovered, these sorting mechanisms are likely to be important for cell development and differentiation. The knockout mouse model for CBF-A/hnRNPab already displays brain developmental issues (Sinnamon et al., 2012). Further, in the same knockout mouse model spermatogenesis is impaired (Fukuda et al., 2013). We predict that tissue-specific factors that differentially interact with the CBF-A/hnRNPab isoforms and RNA sites proximal to the trafficking elements play an important role, contributing to the specific function of CBF-A/hnRNPab in cell specification and development. This is a possible general scenario that, in principle, is applicable to how other hnRNP proteins perform specialized tasks in mRNA trafficking.

Future work will need to proceed toward controlled cell differentiation systems in combination with genome-wide analyses to understand how cell development is controlled by CBF-A/hnRNPab. As more molecular mechanisms are being revealed that regulate mRNA biogenesis from the gene to polysomes, systems biology will provide a powerful approach to understand the importance of specific hnRNP proteins in tissue development and differentiation of complex multicellular organisms and how these mechanisms are potentially impaired in human diseases.

## Author Contributions

All authors wrote the paper, read, and approved the final manuscript.

## Conflict of Interest Statement

The authors declare that the research was conducted in the absence of any commercial or financial relationships that could be construed as a potential conflict of interest.
